# Age-related similarities and differences in brain activity underlying reversal learning

**DOI:** 10.3389/fnint.2013.00037

**Published:** 2013-05-30

**Authors:** Kaoru Nashiro, Michiko Sakaki, Lin Nga, Mara Mather

**Affiliations:** ^1^Center for Vital Longevity, University of Texas at DallasDallas, TX, USA; ^2^Davis School of Gerontology, University of Southern CaliforniaLos Angeles, CA, USA

**Keywords:** aging, emotion, memory updating, functional MRI, reversal learning, associative memory

## Abstract

The ability to update associative memory is an important aspect of episodic memory and a critical skill for social adaptation. Previous research with younger adults suggests that emotional arousal alters brain mechanisms underlying memory updating; however, it is unclear whether this applies to older adults. Given that the ability to update associative information declines with age, it is important to understand how emotion modulates the brain processes underlying memory updating in older adults. The current study investigated this question using reversal learning tasks, where younger and older participants (age ranges 19–35 and 61–78, respectively) learn a stimulus–outcome association and then update their response when contingencies change. We found that younger and older adults showed similar patterns of activation in the frontopolar OFC and the amygdala during emotional reversal learning. In contrast, when reversal learning did not involve emotion, older adults showed greater parietal cortex activity than did younger adults. Thus, younger and older adults show more similarities in brain activity during memory updating involving emotional stimuli than during memory updating not involving emotional stimuli.

In everyday life, we often encounter the same objects, people, or events in different contexts, which allows us to learn something new about old information. For example, you may place your car keys at different places throughout the day, requiring you to update your memory of where they are at any given point (i.e., updating key-location associations). Also, you may see your officemate everyday but notice that he is in a bad mood on a particular day based on the changes in his facial expression (i.e., updating person-expression associations). In this paper, we use the term “updating” to refer to new learning of old information that you have previously encountered, as illustrated in the examples above. The ability to update associative information is an important aspect of memory as well as a critical skill for social adaptation, as it allows one to flexibly update information and respond appropriately to a given situation. In younger adults, updating emotionally arousing information has been shown to be more difficult than updating neutral information (Mather and Knight, 2008; Novak and Mather, 2009), and to activate frontopolar/orbitofrontal (OFC) regions more than updating neutral information in working memory (Nashiro et al., 2012b) as well as in long-term memory (Sakaki et al., 2011). However, it has not been clear whether older adults show similar differences across emotional and neutral updating tasks. Given the fact that the ability to update associative information declines with age (Mell et al., 2005; Weiler et al., 2008), it is important to understand whether and how the effects of emotional arousal on memory updating changes with age. As a first step, the current study focused on examining brain mechanisms underlying updating with the presence and absence of emotion in older adults compared with younger adults.

In contrast with its role in new learning, the amygdala may work against updating emotional memories. Much previous work indicates that amygdala activity during initial learning of emotional material is associated with enhanced long-term memory for that information (e.g., Cahill et al., 1996; Hamann et al., 1999; Dolcos et al., 2004; LaBar and Cabeza, 2006; Murty et al., 2010; Kensinger et al., 2011). Similarly, animal studies in long-term memory indicate that the amygdala enhances memory consolidation for emotional events (McGaugh, 2000). However, the same processes that allow the amygdala to help maintain the original representations of emotional memories could make it more difficult to update those representations.

Recent research suggests that frontopolar/OFC regions play a critical role in updating emotional memory by countering amygdala activity. One recent study using a long-term memory paradigm (Sakaki et al., 2011) found that the frontopolar OFC regions showed greater activity while learning new associations for old emotional items than for new emotional items. In addition, they found that the frontal pole had negative correlations with the amygdala when people learned new associations to old emotional items.

Reversal learning tasks are often used to study short-term memory updating (e.g., Kringelbach and Rolls, 2003; Ghahremani et al., 2010). Similar to the findings in long-term memory (Sakaki et al., 2011), our recent study on reversal learning (Nashiro et al., 2012b) revealed that the frontopolar OFC regions showed greater activity while people were engaged in emotional than neutral memory updating. We also found greater negative correlations between the OFC and the amygdala when updating negative associations than when updating neutral associations. These results suggest that the frontopolar OFC helps update old emotional memories by suppressing the amygdala's protection of old representations in both long-term and short-term memory.

Animal experiments using reversal-learning tasks requiring updating stimulus-reward contingencies based on feedback provide further evidence for the opposing roles of the amygdala and OFC. One study (Stalnaker et al., 2007) used a reversal learning task of odor-solution associations and demonstrated that reversal learning was impaired in the OFC-lesioned group but was not affected in the amygdala-lesioned group. Strikingly, damage to both OFC and amygdala did not impair reversal learning compared to a control group without any lesions. The results suggest that the amygdala protects old emotional representations making it hard to update them, whereas the OFC opposes this amygdala effect. Extinction tasks involving long-term memory also require updating of associations and amygdala lesions have been shown to facilitate the extinction of emotional instrumental responses in macaque monkeys, whereas OFC lesions impair extinction (Izquierdo and Murray, 2005).

Since the studies described previously were all conducted with younger humans or on animals, it remains unclear whether these brain mechanisms would also apply to older adults. In general, evidence suggests that the amygdala remains relatively intact in older adults (for review see Nashiro et al., 2012a); however, previous findings regarding age-related changes in the frontopolar OFC are more ambiguous. In terms of age-related structural changes, previous research found age-related volume declines in lateral and orbital frontal gray matter (Tisserand et al., 2002) and in the frontal pole gray matter (Salat et al., 2001; John et al., 2009). In contrast, another study (Salat et al., 2001) found that OFC volume accounted for a larger proportion of prefrontal volume for older adults than for younger adults, suggesting the OFC declines less with age than other prefrontal regions. A study with a particularly large sample of participants (*N* = 883) is consistent with this lack of OFC decline, as negative correlations between age and cortical thickness were seen in lateral and superior prefrontal regions, but not in the medial OFC (Fjell et al., 2009). A functional MRI study (Lamar et al., 2004) examined age differences in OFC function by employing delayed match and non-match to sample tasks previously shown to elicit OFC involvement. They found that younger compared with older adults showed greater activity in the lateral OFC (BA 47), suggesting that age-related alteration in lateral OFC recruitment contributes to older adults' poor performance on the tasks. However, since this study used neutral stimuli, it is unclear whether the same task involving emotional stimuli would also result in age differences in lateral OFC activity. Although not emphasized in their report, the same study also indicated that there were no age-related differences in frontopolar (BA 10) activity during non-match in contrast to match to sample tasks, consistent with the possibility that the frontal pole functions similarly between younger and older adults.

Despite the fact that it remains unclear how age might affect the structure and function of the frontopolar OFC regions and the interactions between these regions and the amygdala, previous behavioral studies suggested that younger and older adults showed similar enhancing and impairing effects of emotional arousal on associative memory and memory updating (Kensinger, 2008; Nashiro and Mather, 2011; Nashiro et al., 2013). Thus, the current study examined whether the brain mechanisms underlying emotional memory updating are also similar between the two age groups.

## Methods

### Participants

Nineteen undergraduates (*M*_age_ = 25.38, age range = 19–35, 11 males, 8 females, *M*_education_ = 15.3) and 22 older adults (*M*_age_ = 68.00, age range = 61–78, 11 males, 11 females, *M*_education_ = 16.4) participated in the study. The results from the younger participants are described in Nashiro et al. (2012b). Participants provided written informed consent approved by the University of Southern California (USC) Institutional Review Board and were paid for their participation. Prospective participants were screened and excluded for any medical, neurological, or psychiatric illness. Two younger and two older adults were excluded from all analyses due to very poor task performance (their number of errors or number of no responses was greater than 3 standard deviations above the mean). One older adult was excluded due to indications of a previous stroke, which was unknown to the participant prior to the study.

### Materials

The face stimuli were color images obtained from the FACES database developed at the Max Planck Institute for Human Development (Ebner et al., 2010), which included young, middle-aged, and older adults' female and male faces.

Thirty individuals' faces, which had neutral, happy, angry, and eyeglasses versions, were used in the main experiment. These faces were grouped into 15 pairs of two faces from the same age group (i.e., five pairs of younger faces, five pairs of middle-aged faces, and five pairs of older faces), and the gender of each pair was always the same (i.e., male–male, female–female pairs). One out of five pairs in each age category was randomly selected and assigned to each participant, resulting in three pairs from different age groups being used for each participant. Which of the three pairs were used for which of the three conditions was randomly determined for each participant. Gender of face pairs were counterbalanced across participants, such that half of the participants saw two female pairs and one male pair while the other half saw one female pair and two male pairs. Which face in a pair appeared on the left vs. right side of the screen was randomized on each trial.

### Behavioral procedures

We used a reversal learning task, which is designed to test the ability to update associative information in working memory. The details of the procedure were described in our recent publication (Nashiro et al., 2012b) and will be briefly summarized below.

**Figure 1:**
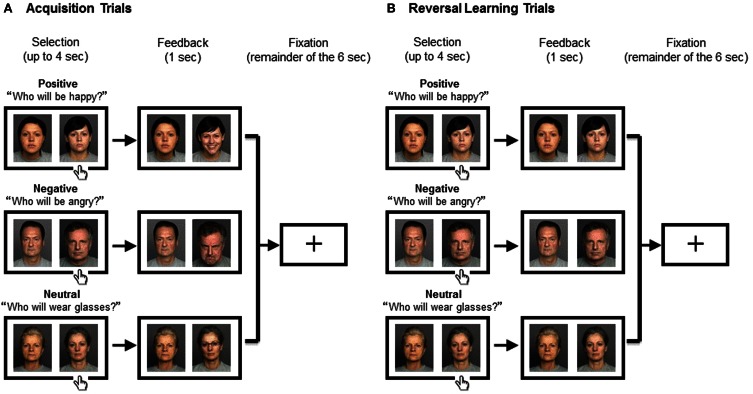
**Experimental Procedure.** The positive (top), negative (middle), or neutral blocks (bottom) were assigned to the participant in a random order. The two people were randomly assigned to the right or the left of the screen. The trial began with a presentation of two people displaying neutral expressions during which the participant had to select one person by pressing a key. Feedback was presented for 1 s, which was followed by a fixation cross for the remainder of the 6 s. **(A)** In Acquisition Trials where the response was correct, the selected face changed (into a happy face, angry face, or face with eyeglasses, respectively), while the other face remained neutral. **(B)** In Reversal Learning Trials where the response was incorrect, both of the faces remained neutral. Across conditions, the task for the subject was to keep track of the correct person because it switched mid-game. The correct person changed after several (between three and six) consecutive correct trials; the number of trials before the change was unknown to the subject.

The main experiment consisted of positive, negative, and neutral blocks, the order of which was randomized across the participants. At the beginning of each block, a prompt appeared; “Who is happy?” “Who is angry?” or “Who wears glasses?” in the positive, negative, or neutral conditions respectively (Figure 1). Each trial lasted for 6 s, which consisted of (1) selection, (2) feedback, and (3) fixation periods. (1) Selection period: two neutral faces were presented with a white background. Participants were asked to select one face with the target characteristics (happy, angry, or eyeglasses) by pressing a key corresponding to the left or right side of the screen. (2) Feedback period: immediately after their response, feedback was presented for 1 s on a gray background. If the response was correct, the selected face changed (into a happy face, angry face, or face with eyeglasses), while the other face remained neutral. If the response was incorrect, both of the faces remained neutral. When the participant did not respond within 4 s, the warning “please respond faster” was displayed in the center of the screen instead of feedback faces. (3) Fixation period: the trial ended with a fixation cross for the remainder of the 6 s.

After 3–6 consecutive correct responses, which face was correct was reversed. We used a randomized variable acquisition criterion of 3–6 trials correct before each reversal in order to keep the participants engaged in the task as well as making which trials involved a reversal unknown to the participants until they received feedback. They were asked to keep track of the correct face and change their answers as soon as noticed the switch.

### Trial modeling

Each trial was categorized as one of three trial types: reversal, acquisition, and other. “Reversal” described individual trials where the participant selected the previously correct person, but because this answer was no longer correct, the feedback was a neutral face expression of the selected person. Reversal trials were defined so that they were always followed by a response shift in the next trial; thus, trials where the participant selected the previously correct person, but did not change their response in a subsequent trial were not included. This categorization allowed us to capture brain activity when the participant made a final error immediately before switching their response. It should be noted that there were no differences in terms of the perceptual properties or the stimulus emotionality across positive, negative, and neutral conditions during the reversal trials since participants viewed two neutral faces during reversal in all conditions. “Acquisition” included trials in which the participant's correct choices of a particular person led to a change in the face (i.e., happy face, angry face, or face appearing with eyeglasses). The first trial of each condition was modeled as “other” (regardless of whether the subject made a correct or incorrect choice), as these trials required subjects to guess and do not reflect learning (or failure of learning) of previous associations. The rest of the trials, which did not fall into the categories of reversal or acquisition trials, were also aggregated as “other.” For example, “other” includes trials where the participant chose incorrect faces before reaching the criterion (3–6 consecutive correct responses) or trials where the participant failed to respond within 4 s.

### Functional MRI data acquisition and preprocessing

Imaging was conducted with a 3 T Siemens MAGNETOM Trio scanner with a 12-channel matrix head coil at the University of Southern California Dana and David Dornsife Neuroimaging Center. The imaging parameters were repetition time (TR) = 2000 ms, echo time (TE) = 25 ms, slice thickness = 3 mm, interslice gap = 0 mm, flip angle (FA) = 90°, final voxel dimension = 3 × 3 × 3 mm, and field of view (FOV) = 192 × 192 mm. Data preprocessing were performed using FMRIB's Software Library (FSL; www.fmrib.ox.ac.uk/fsl), which included motion correction with MCFLIRT, spatial smoothing with a Gaussian kernel of full-width half-maximum 5 mm, high-pass temporal filtering equivalent to 100 s, and skull stripping of structural images with BET. MELODIC ICA (Beckmann and Smith, 2004) was used to remove noise components. Registration was performed with FLIRT; each functional image was registered to both the participant's high-resolution brain-extracted structural image and the standard Montreal Neurological Institute (MNI) 2-mm brain.

### fMRI data analyses

#### Whole-brain analysis

For each reversal trial for each participant, stimulus-dependent changes in BOLD signal were modeled with regressors for feedback and fixation periods. We expected that on reversal trials, participants should try to update associations between face and outcomes not only during the brief feedback period but also during the subsequent fixation. Thus, signal from the feedback and fixation periods were averaged for each valence condition to capture more reliable BOLD signal for reversal learning. The selection period (the initial presentation of two neutral faces) was modeled as the baseline level of activity and therefore was not included as a regressor. Motion regressors were also included. “Acquisition” and “other” trials were also modeled. The regressors were convolved with a double-gamma hemodynamic response function and temporal filtering was applied as well. Temporal derivatives of each of the regressors were also included.

Whole-brain analyses were conducted using FSL FEAT v. 5.98 (FMRIB's Software Library, www.fmrib.ox.ac.uk/fsl). *Z* (Gaussianised T/F) statistic images were thresholded at the whole-brain level using clusters determined by *Z* > 2.3 and a (corrected) cluster significance threshold of *p* = 0.05 (Worsley, 2001) unless otherwise noted. Locations reported by FSL were converted into Talairach coordinates by the MNI-to-Talairach transformation algorithm (Lancaster et al., 2007). These coordinates were used to determine the nearest gray matter using the Talairach Daemon version 2.4.2 (Lancaster et al., 2000).

#### Regions-of-interest (ROI) analyses

Previous research suggests that the lateral OFC, in particular, plays an important role in reversal learning (Hampshire and Owen, 2006; O'Doherty et al., 2001). Therefore, we performed ROI analyses to examine whether this OFC sub-region shows different activities in reversal learning across the conditions. The left and right lateral OFC were structurally defined using UCLA's Laboratory of Neuro Imaging LPBA40 atlas (Shattuck et al., 2008), set at a 0.5 probabilistic threshold.

Given past findings that the amygdala plays a role in reversal learning (Izquierdo and Murray, 2005; Stalnaker et al., 2007) and our interest in how emotional reversal learning differs from non-emotional reversal learning, we performed ROI analyses for the left and right amygdala. The amygdala was segmented from each participant's high resolution structural scan using FreeSurfer (surfer.nmr.mgh.harvard.edu) and FSL FAST (FMRIB's Software Library, www.fmrib.ox.ac.uk/fsl). For each hemisphere for each participant, we examined the results from each segmenting software and selected the one judged as more accurate for further manual correction. Next, manual correction of this selected ROI was carried out and erroneous voxels in non-amygdala regions (e.g., hippocampus, white matter) were removed. For both ROI analyses, FSL Featquery was used to extract percent signal change values.

#### Functional connectivity analyses

The structurally defined amygdala (defined as described above) served as a seed region. To examine functional connectivity, we applied a beta series correlation analysis, which has been found to be an effective measure for functional connectivity (Rissman et al., 2004; Gazzaley et al., 2005). We combined feedback and fixation periods for each reversal trial for each participant. Stimulus-dependent changes in BOLD signal were then modeled with regressors for the “feedback and fixation” period for each reversal trial, while the selection period served as baseline. This allowed us to obtain trial-to trial parameter estimates of reversal-specific activity. First, a new GLM design file was constructed where each reversal trial was coded as a unique covariate, resulting in up to 39 independent variables (the maximum number of reversal trials achieved by participants across all three conditions). To reduce the confounding effects of the global signal change, the mean signal level over all brain voxels was calculated for each time point and was used as a covariate. The model also involved additional nuisance regressors for acquisition and “other” trials. Second, the least squares solution of the GLM yielded a beta value for each reversal trial for each individual participant. These beta values were then sorted by conditions. Third, mean activity (i.e., mean parameter estimates) was extracted for each individual reversal trial from a seed region. Fourth, for each condition, we computed correlations between the seed's beta series and the beta series of all other voxels in the brain, thus generating condition-specific seed correlation maps. Correlation magnitudes were converted into *z*-scores using the Fisher's *r*-to-*z* transformation. Condition-dependent changes in functional connectivity were assessed using random-effects analyses, which were thresholded at the whole-brain level using clusters determined by *Z* > 2.3 and a (corrected) cluster significance threshold of *p* = 0.05.

## Results

### Behavioral results

We found no significant main effects of group or condition, and no interactions between group and condition in any measure, as reported below (also see Table 1).

**Table 1:** **Behavioral results showing no significant differences between group and conditions**.

**Condition**	**Reversal error**		**Other error**		**Acquisition trials**		**Reaction time (ms)**	
	**Younger**	**Older**	**Younger**	**Older**	**Younger**	**Older**	**Younger**	**Older**
Positive	10.41 (0.49)	10.63 (0.46)	4.00 (0.68)	4.11 (0.64)	5.18 (0.25)	4.96 (0.23)	740	760
Negative	10.82 (0.44)	10.68 (0.42)	3.24 (0.67)	3.84 (0.63)	4.93 (0.14)	4.72 (0.13)	700	740
Neutral	10.94 (0.50)	10.21 (0.47)	3.47 (0.92)	5.16 (0.87)	4.90 (0.23)	5.12 (0.22)	720	830

The table shows the means of the total number of reversal errors (A), the total number of other errors (B), the average number of trials before reaching the acquisition criteria (C), and the average reaction time immediately after reversal trials (D). There were no significant differences between groups and among conditions, and no significant interactions in any measure.

The errors made in the first trial of each condition were excluded, as those were guessing errors and were not due to failure of learning previous associations. The rest of the errors were divided into two types: reversal and other. The total number of reversal errors was calculated for each condition. A 2 (group: younger, older) × 3 (conditions: positive, negative, neutral) mixed analysis of variance (ANOVA) revealed no significant findings. There were no significant differences between groups, *F*_(1, 34)_ = 0.14, *p* = 0.71, and conditions, *F*_(2, 68)_ = 0.39, *p* = 0.68. No significant interaction between group and condition was found, *F*_(2, 68)_ = 1.50, *p* = 0.23. Similarly, there were no significant findings in the total number of other errors between groups, *F*_(1, 34)_ = 0.74, *p* = 0.40, and between conditions, *F*_(2, 68)_ = 1.71, *p* = 0.19. No significant interaction between group and condition was found, *F*_(2, 68)_ = 1.79, *p* = 0.17. The average number of trials before reaching the acquisition criteria (3–6 consecutive correct responses) was calculated for each condition. There were no significant differences between groups, *F*_(1, 34)_ = 0.09, *p* = 0.77, and between conditions, *F*_(2, 68)_ = 1.45, *p* = 0.24, and there was no significant interaction between group and condition, *F*_(2, 68)_ = 1.36, *p* = 0.26. Lastly, to examine how quickly participants responded to the correct face after making reversal errors, the average reaction time for trials immediately after reversal trials was calculated for each condition. There were no significant differences between groups, *F*_(1, 34)_ = 2.24, *p* = 0.14, and between conditions, *F*_(2, 68)_ = 2.89, *p* = 0.06, and no significant interaction between group and condition, *F*_(2, 68)_ = 2.01, *p* = 0.14.

### fMRI results

First, we contrasted brain activity during reversal and acquisition in order to examine the brain regions that are more important for reversal learning than acquisition. For the rest of the analyses, we contrasted brain activity during reversal learning in different conditions. In these contrasts across conditions, there were no differences in the perceptual properties or the visual stimulus emotionality, as all reversal trials involved seeing neutral faces.

### Common activation between younger and older adults

#### Brain regions showing greater activity during reversal than acquisition in both groups

Reversal-acquisition contrasts were first performed at the single-subject level for all conditions. These were then entered into a second-level random-effects analysis to determine the brain areas that showed significantly greater activity in reversal than acquisition trials across subjects. Collapsed across groups and conditions, reversal compared with acquisition trials produced increased activity in inferior frontal gyrus/OFC (BA 47), frontal pole (BA 10), inferior frontal gyrus (BA 9), anterior cingulate cortex (BA 24 and 32), and insula (BA 13). Furthermore, putamen, caudate, thalamus, posterior cingulate cortex (BA 23 and 30), precentral gyrus (BA 6), superior temporal gyrus (BA 22), and inferior parietal lobule (BA 40) showed increased activity in reversal than acquisition trials. Thus, consistent with previous research (e.g., Kringelbach and Rolls, 2003; Rolls and Grabenhorst, 2008; Ghahremani et al., 2010; Tsuchida et al., 2010), the OFC and the frontal pole showed greater activity during reversal than acquisition trials, indicating these regions were involved in reversal learning. In a second level analysis, we also examined group differences using independent *t*-tests; but found no differences in either younger-older or older-younger contrasts, suggesting that the two groups produced similar activity during reversal compared with acquisition trials.

#### Brain regions showing different activity during emotional vs. neutral reversal learning in both groups

**Figure 2:**
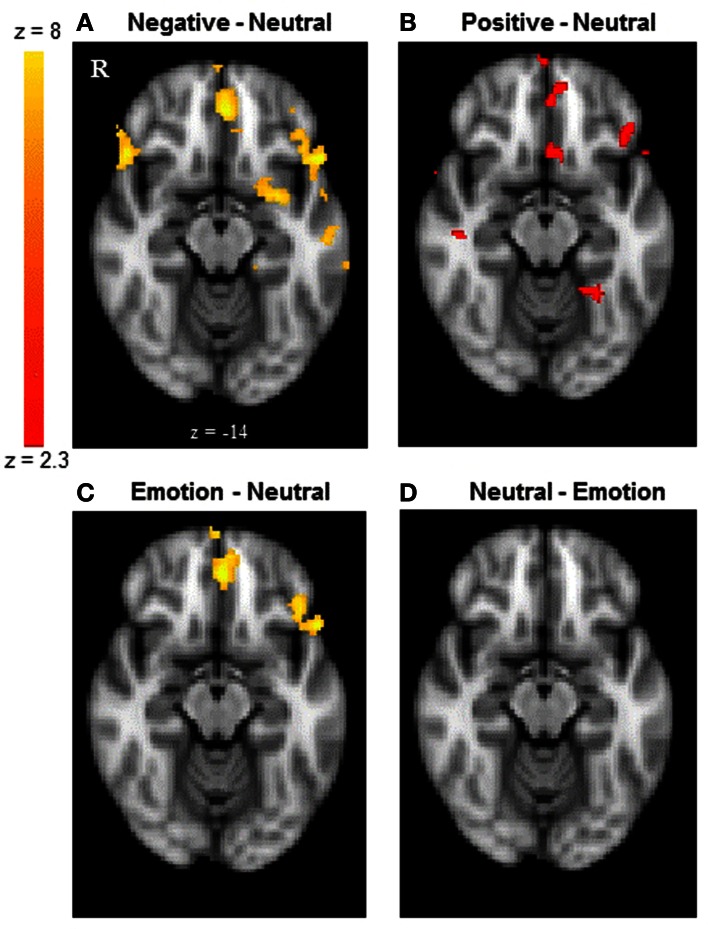
**The frontal pole and OFC showed similar activity between younger and older adults.** The results shown here are collapsed across groups. **(A)** The frontal pole and OFC showed greater activity when participants reversed negative associations than neutral associations. **(B)** The positive-neutral contrast also showed a similar pattern of frontopolar OFC activity when a lower threshold (a voxel-threshold of *z* = 2.3) was used. Although the low-threshold map should be interpreted with caution, it provides useful information about the similarities between the positive and negative conditions in contrast with the neutral condition. **(C)** When positive and negative conditions were combined, the emotion condition showed greater activity in the frontal pole and OFC than did the neutral condition, **(D)** whereas the reverse contrast showed no significant findings. The images were thresholded at the whole-brain level using clusters determined by *z* > 2.3 and a (corrected) cluster significance threshold of *p* = 0.05, except for panel **(B)**.

Next, we examined whether reversal learning in the positive and negative emotion conditions produced different patterns of brain activity than reversal learning in the neutral condition across younger and older adults. Thus, the analyses below collapsed across groups. The whole-brain analysis revealed greater activity in the negative than neutral conditions in inferior frontal gyrus/OFC (BA 47, Figure 2A), frontal pole (BA 10), superior frontal gyrus (BA 9), and anterior cingulate (ACC; BA 32). Other regions showing significant differences in the negative-neutral contrast are reported in Table 2. There were no significant findings in other contrasts (negative-positive, positive-negative, positive-neutral, neutral-positive, neutral-negative). However, when we used a lower threshold (a voxel-threshold of *z* = 2.3), the positive-neutral contrast yielded similar results to the ones in the negative-neutral contrast. Compared with the neutral condition, the positive condition produced greater activity in inferior frontal gyrus/OFC (BA 47; Figure 2B), frontal pole (BA 10), and ACC (BA 32). Although these results based on use of a lower threshold should be interpreted with caution, they provide useful information about the similarities between the positive and negative conditions in contrast with the neutral condition. Next, the positive and negative conditions (together called the emotion condition) were combined and contrasted against the neutral condition. The emotion condition yielded greater activity in areas including inferior frontal gyrus/OFC (BA 47), frontal pole (BA 10), and ACC (BA 32) than did the neutral condition, whereas the reverse contrast showed no significant findings (Table 2; Figures 2C,D).

**Table 2:** **Brain activity showing significant differences between conditions during reversal learning in younger and older adults**.

**Area**	**H**	***BA***	**MNI**			**Talairach**			***Z*-max**
			***x***	***y***	***z***	***x***	***y***	***z***	
**NEGATIVE > NEUTRAL**									
Putamen	L		−26	−2	6	−25	−4	9	3.71
Putamen	L		−32	−2	4	−31	−4	7	3.41
Inferior frontal gyrus	L	47	−52	20	−14	−49	18	−7	3.34
Middle occipital gyrus	L	18	−22	−94	14	−22	−90	8	3.45
Fusiform gyrus	L	37	−48	−54	−2	−46	−52	−3	3.45
Middle occipital gyrus	L	19	−26	−92	16	−25	−89	10	3.35
Cuneus	L	17	−8	−84	12	−9	−81	7	3.2
Posterior cingulate	L	31	−6	−38	32	−7	−40	29	3.2
Culmen	L		−4	−72	−2	−5	−69	−4	3.16
Anterior cingulate	L	32	−2	44	−20	−3	41	−10	3.37
Superior frontal gyrus/frontal pole	R	9/10	4	70	18	3	62	26	3.29
Anterior cingulate	L	32	−2	44	−10	−3	40	−1	3.29
Inferior frontal gyrus	R	47	56	22	−8	51	19	0	3.25
Inferior frontal gyrus	R	47	52	22	−14	47	20	−6	3.23
Claustrum	R		38	16	−8	34	14	−1	3.03
Superior temporal gyrus	R	42	66	−30	16	60	−31	17	3.19
Superior temporal gyrus	R	42	58	−34	12	52	−35	13	3.07
Middle temporal gyrus	R	21	66	−22	−6	60	−22	−2	3.02
Thalamus	L		−6	−24	−4	−7	−24	−2	3.53
Thalamus	L		−6	−24	0	−7	−24	2	3.4
Thalamus	L		−8	−22	4	−9	−23	6	3.29
Positive > Neutral	No significant results								
Negative > Positive	No significant results								
Positive > Negative	No significant results								
Neutral > Negative	No significant results								
Neutral > Positive	No significant results								
**EMOTION > NEUTRAL**									
Fusiform gyrus	L	37	−48	−54	−2	−46	−52	−3	3.37
Fusiform gyrus	L	37	−58	−56	6	−55	−54	4	3.24
Middle temporal gyrus	L	21	−68	−20	−10	−64	−19	−8	3.15
Lingual gyrus	L		−6	−84	8	−7	−81	4	3.33
Cuneus	R	18	6	−78	14	4	−76	10	3.06
Culmen	L		−4	−72	−2	−5	−69	−4	3.02
Inferior frontal gyrus	L	47	−36	32	−4	−34	29	3	3.31
Inferior frontal gyrus	L	47	−52	20	−14	−49	18	−7	3.28
Inferior frontal gyrus	L	47	−44	28	−12	−42	26	−5	3.22
Anterior cingulate	L	32	−2	44	−20	−3	41	−10	3.57
Anterior cingulate	L	32	−2	46	−12	−3	42	−3	3.4
Frontal pole	R	10	2	66	−12	1	61	−1	3.19
Neutral > Emotion	No significant results								

#### ROI analysis for the OFC

**Figure 3:**
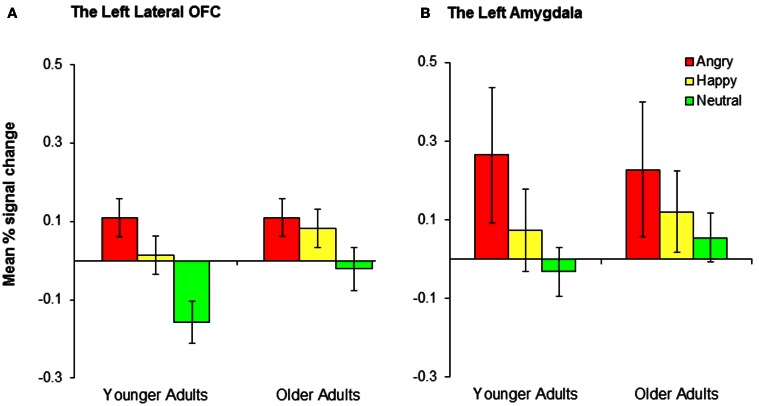
**Younger and older adults showed similar patterns of activity in (A) the left lateral OFC and (B) the left amygdala across conditions.** Collapsed across groups, participants showed significantly greater activity in the negative than neutral conditions and the positive than neutral conditions, although the differences in the positive-neutral contrast for the left amygdala did not achieve significance.

A 2 (group: younger, older) × 3 (condition: positive, negative, neutral) mixed analysis of variance (ANOVA) was performed on the percent signal change from the left and right lateral OFC. There was a significant effect of condition in the left lateral OFC, *F*_(2, 68)_ = 11.08, *MSE* = 0.03, *p* < 0.001, η_*p*2_ = 0.25, whereas there was no significant effect of group (*p* = 0.19) and no significant interaction between group and condition (*p* = 0.30). *Post-hoc t*-tests suggest that the left lateral OFC showed significantly greater activity in the negative than the neutral conditions, *t*_(35)_ = 4.46, *p* < 0.001, and in the positive than the neutral conditions, *t*_(35)_ = 3.16, *p* = 0.003, whereas there was no significant difference between the negative and the positive conditions (*p* = 0.18; see Figure 3A). These results suggest that the left lateral OFC is more involved in emotional reversal learning than in neutral reversal learning in both younger and older adults. For the right OFC, there was a significant effect of group (*M*_younger_ = −0.002; *M*_older_ = 0.10), *F*_(1, 34)_ = 4.38, *MSE* = 0.07, *p* = 0.04, η_*p*2_ = 0.11, but no other findings. Across conditions, older adults recruited the right lateral OFC more than did younger adults, but no significant effects of condition were found.

#### ROI analysis for the amygdala

We conducted 2 (group: younger, older) × 3 (conditions: conditions: positive, negative, neutral) mixed ANOVAs on the percent signal change from the left and right amygdala. There was a significant effect of condition in the left amygdala, *F*_(2, 68)_ = 3.48, *MSE* = 0.15, *p* = 0.04, η_*p*2_ = 0.09. A *post-hoc t*-test indicated that the left amygdala showed significantly greater activity in the negative than the neutral conditions, *t*_(35)_ = 2.71, *p* = 0.01 (Figure 3B). The right amygdala showed a similar pattern, although the result was only marginally significant, *F*_(2, 68)_ = 2.94, *MSE* = 0.15, *p* = 0.06, η_*p*2_ = 0.08. The right amygdala also showed significantly greater activity in the negative than the neutral conditions, *t*_(35)_ = 2.19, *p* = 0.04. No age group differences were found in any of these analyses.

#### Functional connectivity analysis with the amygdala as a seed region

A whole-brain connectivity analysis with the left amygdala as a seed region was conducted for each condition. The negative condition produced a significant inverse correlation between the left amygdala and the right middle frontal gyrus/frontal pole (BA 9, 10) whereas such negative correlations were not observed in the positive and neutral conditions (Table 3). The same analysis with the right amygdala as a seed region did not show negative correlations with the frontopolar regions in any of the conditions. No age differences were found in any of these analyses.

**Table 3:** **Brain regions showing negative connectivity with the left amygdala across groups**.

**Area**	**H**	***BA***	**MNI**			**Talairach**			***Z*-max**
			***x***	***y***	***z***	***x***	***y***	***z***	
**NEGATIVE**									
Inferior parietal lobule	R	40	48	−48	44	44	−49	40	3.97
Inferior parietal lobule	R	40	48	−50	52	44	−52	47	3.87
Inferior parietal lobule	R	40	40	−44	38	37	−45	35	3.86
Middle frontal gyrus	R	9	42	18	38	39	13	39	5.16
Middle frontal gyrus	R	9	44	24	36	41	19	37	4.68
Frontal pole	R	10	32	60	20	29	54	25	4.56
Precuneus	R	7	8	−72	50	6	−72	44	4.61
Precuneus	R	7	6	−56	64	4	−58	57	4.12
Precuneus	R	7	−18	−78	50	−19	−78	43	3.46
**POSITIVE**									
Precuneus	R	7	18	−78	52	16	−78	45	3.92
Precuneus	R	31	6	−74	28	4	−73	24	3.76
Precuneus	R	7	14	−78	52	12	−78	45	3.74
**NEUTRAL**									
Precuneus	R	7	−4	−80	50	−5	−80	43	4.18
Cuneus	R	19	2	−82	44	0	−81	38	4.08
Cuneus	R	18	2	−76	36	0	−75	31	3.84

### Age-related differences in brain activity during reversal learning

**Figure 4:**
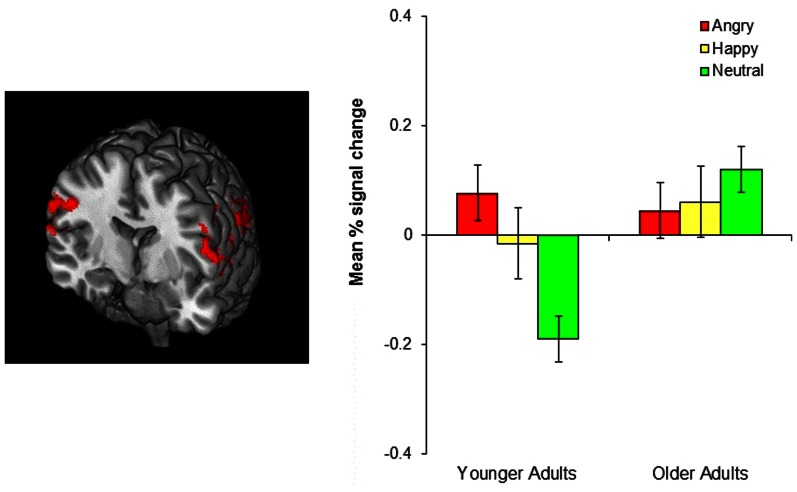
**The left image shows age-related differences in the parietal cortex in the neutral condition.** Older adults showed greater parietal cortex activity than did younger adults. The image was threshholded at the whole-brain level using clusters determined by *z* > 2.3 and a (corrected) cluster significance threshold of *p* = 0.05. The bar graph on the right shows the mean % signal change in the bilateral parietal cortex, revealing that younger adults showed greater parietal cortex activity in the negative and positive than in the neutral conditions (*p* = 0.001, *p* = 0.034, respectively) whereas older adults showed no difference across conditions.

Although no age-related differences were observed in the frontopolar OFC and the amygdala, the whole-brain analyses for the negative-neutral and neutral-negative contrasts revealed age differences in the inferior parietal lobule (BA 40), superior temporal gyrus (BA 39, 42), precuneus (BA 7), precentral gyrus (BA 6), and postcentral gyrus (BA 3). Similarly, significant age differences were found for the emotion-neutral and neutral-emotion contrasts in inferior parietal lobule (BA 40) and superior temporal gyrus (BA 39, 42). To better identify the nature of these age-by-emotion interactions, we directly compared younger and older adults separately for each of the three emotion conditions. The positive and negative conditions did not produce significant age differences in any of the brain regions. In contrast, in the neutral condition, older adults showed greater activity in the inferior parietal lobule (BA 40), superior temporal gyrus (BA 41, 42), precentral gyrus (BA 4, 6), and superior occipital gyrus (BA 19) than did younger adults (see Figure 4).

## Discussion

This study aimed to examine whether brain mechanisms underlying emotional memory updating would be similar between younger and older adults. Our results demonstrated that across age groups, emotional reversal learning produced greater activity in the OFC and the frontal pole than did neutral reversal learning. Importantly, frontopolar/OFC activity did not significantly differ between younger and older adults during emotional reversal learning. Consistent with previous research suggesting that the amygdala remains relatively intact with age, both groups showed significantly greater activity in the amygdala during negative than neutral reversal learning. Furthermore, both groups showed negative correlations between the amygdala and the middle frontal gyrus/frontal pole (BA 9/10) during negative reversal learning. Past research revealed that the frontal pole has negative correlations with the amygdala when updating old emotional memories (Finger et al., 2008; Sakaki et al., 2011). Our findings are consistent with those previous results and suggest that the frontopolar OFC helps update old associations by down-regulating the amygdala's protection of old representations (Schoenbaum et al., 2007; Stalnaker et al., 2007). It is interesting that similar negative correlations have been seen across studies using different types of associations to be updated, including both item-context associations (as in Sakaki et al., 2011) and within-item feature associations (as in the current study). This suggests that, despite differences in whether the hippocampus, perirhinal cortex, or parahippocampal cortex is most critical for the specific type of binding involved (Diana et al., 2010; Staresina et al., 2011), the amygdala's involvement in updating associations to emotional memories is modulated by frontopolar OFC. Importantly, our results suggest that this mechanism applies to both younger and older adults. The similarity of the relationship between the frontopolar OFC and amygdala among younger and older adults is consistent with evidence that these regions are relatively well-maintained in aging (Salat et al., 2001; Fjell et al., 2009; Nashiro et al., 2012a).

In contrast with emotional reversal learning, neutral reversal learning produced age-related differences in the parietal cortex, such that older adults showed greater parietal cortex activity than did younger adults. This age difference seems to be due to the fact that younger adults recruited this region only for emotional reversal learning, but not for neutral reversal, whereas older adults showed similar parietal cortex activation across all types of reversal learning (see the bar graph in Figure 4). This was indicated by significantly greater parietal cortex activity in both the negative and positive conditions than the neutral condition in younger adults, while there was no difference between conditions in older adults. Previous research suggests that the ventral parietal cortex, which showed the most age differences during neutral reversal, reflects bottom-up attention processes elicited by the retrieval cues or by behaviorally relevant stimuli, especially when they are unexpected (Corbetta and Shulman, 2002; Cabeza et al., 2008, 2011). Thus, one possibility is that younger adults paid greater attention to the cues that signaled emotional reversals than the cues indicating neutral reversals, perhaps due to the fact that reversing emotional associations was harder than reversing neutral associations. This is in line with previous evidence suggesting that emotional information is more difficult to update than neutral information (Mather and Knight, 2008; Novak and Mather, 2009). Older adults, on the other hand, might have found emotional and neutral reversals equally difficult resulting in similar level of attention to both types of cues. However, it remains unclear why older adults did not show greater parietal cortex activity in the emotion than the neutral conditions, an issue that needs to be addressed in future studies.

It is unclear why we did not observe negative correlations between the amygdala and the frontopolar regions in the positive condition, unlike those seen in the negative condition. One possible explanation is that positive reversal learning did not evoke as strong an emotional response as did negative reversal learning; therefore, reversals of positive associations required less frontal involvement to modulate old representations in the amygdala than did reversals of negative associations. In fact, our ROI results suggest that bilateral amygdala showed less activity during positive than negative reversal learning in both groups (albeit non-significantly so); this might suggest that positive reversal learning requires fewer resources to down-regulate the amygdala than does negative reversal learning.

In summary, the current study provides new information about age-related similarities and differences in the brain mechanisms of memory updating. Our results suggest that younger and older adults activate similar brain regions during emotional (in contrast with neutral) reversal learning. This is consistent with previous findings suggesting that the effects of emotional arousal on memory remain similar between younger and older adults. In addition, we found age group differences in parietal cortex activity only during neutral memory updating. Future studies should investigate the nature of this age difference. This line of research is particularly important for older adults who experience daily challenges in memory updating, as it may help us distinguish when and how emotion benefits or impairs new learning as well as develop strategies to reduce age-related declines in this cognitive domain.

### Conflict of interest statement

The authors declare that the research was conducted in the absence of any commercial or financial relationships that could be construed as a potential conflict of interest.
